# Authenticity of raspberry flavor in food products using SPME‐chiral‐GC‐MS

**DOI:** 10.1002/fsn3.296

**Published:** 2015-10-26

**Authors:** Anne‐Mette S. Hansen, Henrik L. Frandsen, Arvid Fromberg

**Affiliations:** ^1^ National Food InstituteTechnical University of DenmarkMørkhøj Bygade 19SøborgDK‐2860Denmark

**Keywords:** Authenticity, chiral‐GC‐MS, enantioselective GC, raspberry flavor, SPME, *α*‐ionone

## Abstract

A fast and simple method for authenticating raspberry flavors from food products was developed. The two enantiomers of the compound (E)‐*α*‐ionone from raspberry flavor were separated on a chiral gas chromatographic column. Based on the ratio of these two enantiomers, the naturalness of a raspberry flavor can be evaluated due to the fact that a natural flavor will consist almost exclusively of the R enantiomer, while a chemical synthesis of the same compound will result in a racemic mixture. Twenty‐seven food products containing raspberry flavors where investigated using SPME‐chiral‐GC‐MS. We found raspberry jam, dried raspberries, and sodas declared to contain natural aroma all contained almost only R‐(E)‐*α*‐ionone supporting the content of natural raspberry aroma. Six out of eight sweets tested did not indicate a content of natural aroma on the labeling which was in agreement with the almost equal distribution of the R and S isomer. Two products were labeled to contain natural raspberry flavors but were found to contain almost equal amounts of both enantiomers indicating a presence of synthetic raspberry flavors only. Additionally, two products that were labeled to contain both raspberry juice and flavor showed equal amounts of both enantiomers, indicating the presence of synthetic flavor.

## Introduction

Authenticity of food products is often used as a parameter of quality in marketing. Food products containing only natural ingredients are preferred by many consumers who are willing to pay a higher price for such products. Natural aroma components are often more expensive than their synthetic equivalent making counterfeit an economical benefit. Counterfeit of aroma components is misleading to the consumers and is illegal according to European legislation (European Parliament 2008) where the term “Natural” can be used only for flavors containing exclusively natural flavoring substances. A natural flavor should be obtained by appropriate physical, enzymatic, or microbiological processes from the material of vegetable, animal, or microbiological origin (European Parliament 2008).

The flavor of raspberries consist of many different aroma compounds all contributing more or less to the characteristic perception of raspberries. Malowicki et al. investigated the volatile composition of raspberries following stir bar sorptive extraction and identified some 30 compounds, including (Z)‐hexenol, hexanal, (E)‐2‐hexenal, 2‐heptanone, *δ*‐octalactone, *δ*‐decalactone, geraniol, *α*‐ionone, *β*‐ionone, and terpinen‐4‐ol as the major constituents (Malowicki et al. 2008a,b). Some of the flavoring substances are chiral and the enantiomeric composition in raspberry extracts was characterized by chiral GC‐MS, *α*‐ionone occurs mainly in the R‐form (97–100%), whereas *δ*‐octalactone, *δ*‐decalactone, and terpinen‐4‐ol occurs mainly in the S‐form (80–100%) (Malowicki et al. 2008a,b). Also, Werkhoff et al. (1991) found an enantiomeric composition of *α*‐ionone in raspberry extract sampled by a head space of 99.9% R‐form and 0.1% S‐form (Werkhoff et al. 1991). The enantiomeric composition of *α*‐ionone and *δ*‐decalactone in raspberry extract was by chiral GC determined to 98–100% R‐form for ionone and to 98–100% S‐form for decalactone (Casabianca and Graff 1994). The enantiomeric composition of a number of chiral 4‐alkylated‐*γ*‐lactones from C_5_ to C_12_ was determined in extracts from apricot, mango, passion fruit, peach, raspberry and strawberry (Bernreuther et al. 1989;. Guichard et al. 1990). For all fruits, there seemed to be a preponderance of the R‐form of the longer chained *γ*‐lactones > C_8_ whereas for the shorter chained *γ*‐lactones some fruits had preponderance of the R‐form, some of the S‐form and some hardly showed enantiomeric excess. In both orange flowers and unifloral orange honey, the enantiomeric composition of linalool was 87–91% of the (+) form and 9–13% of the (−) form indicating the usefulness of chiral analysis in the authenticity assessment of unifloral honey (Verzera et al. 2014).

Ravid et al. (2010) assessed the authenticity of natural fruit compounds in food and beverages, using head space SPME chiral GC‐MS. The enantiomeric composition of linalool, linalyl acetate, and limonene were characterized in bergamot oil, *γ*‐decalactone, and *γ*‐undecalactone in peach and nectarine products, *γ*‐lactones in passion fruit products and *α*‐ionone in raspberry products. The study showed that (E)‐*α*‐ionone in raspberry is efficiently adsorbed on an SPME fiber and occur almost exclusively as the R enantiomer, whereas (E)‐*α*‐ionone originating from chemical synthesis is a racemic mixture containing almost equal amounts of both enantiomers. In raspberries, the biosynthesis of (E)‐*α*‐ionone is catalyzed by stereospecific enzymes leading to a preponderance of (R)‐(E)‐*α*‐ionone of more than 99% (Ravid et al. 2010). Consequently, the presence of (S)‐(E)‐*α*‐ionone could be an indicator of adulteration with artificial aroma components (Aprea et al. 2009; Taylor and Linforth 2010).

Head space SPME is an attractive method to isolate and concentrate volatile compounds from complex samples such as foods because only little sample pretreatment is required. In this study, we investigated three different fiber coatings: divinylbenzene/polydimethylsiloxane (DVB/PDMS), carboxen/polydimethylsiloxane (CAR/PDMS), and divinylbenzene/carboxen/polydimethylsiloxane (DVB/CAR/PDMS) for the capabilities to extract volatile compounds, from raspberry aroma and in particular (E)‐*α*‐ionone, as this compound is the major enantiomeric compound bound to the extraction fiber. Furthermore, addition of sodium chloride during head space SPME was investigated for potential improvement of extraction efficiency. Finally, 27 raspberry containing and/or flavored samples of foods, beverages and sweets from the Danish market were analyzed for enantiomer composition of (E)‐*α*‐ionone to investigate whether the labeling were in compliance with the EU regulation. The samples set included sodas and sweets, products which have not previously been investigated for authenticity.

## Materials and Methods

### Samples

Samples of jam, sodas, sweets, dried raspberries, fruit bars, and yoghurts from the Danish retail market were purchased at different retailers, all declared to contain raspberries, natural flavor and/or flavor, see Table 1. Samples of fresh raspberries were obtained from the local retail market. Chemicals: Pure standard of *α*‐ionone was purchased from Sigma‐Aldrich (Steinheim, Germany). Sodium chloride was purchased from Merck (Darmstadt, Germany).

**Table 1:** Sample description and R‐(E)‐*α*‐ionone and S‐(E)‐*α*‐ionone analyses in food and sweet samples from the Danish market

Sample	Fruit content declared	Natural aroma declared	Aroma declared	R‐*α*‐ionone %	S‐*α*‐ionone %	Compliance with EU legislation
Jam #1	Raspberry, 35%			97.1	3.0	Yes
Jam #2	Raspberry, 50%			97.4	2.6	Yes
Jam #3	Raspberry, 50%			96.8	3.2	Yes
Jam #4	Raspberry, 45%			96.3	3.7	Yes
Jam #5	Raspberry, 40%			98.5	1.5	Yes
Jam #6	Raspberry, 45%			97.3	2.7	Yes
Soda #1			x	49.9	50.1	Yes
Soda #2			x	49.8	50.2	Yes
Soda #3		x		100	0	Yes
Soda #4		x		100	0	Yes
Soda #5			x	50.6	49.4	Yes
Dried raspberries #1	Dried fruits			100	0	Yes
Sweet #1, fruit gum			x	49.9	50.1	Yes
Sweet #2, fruit gum			x	49.9	50.1	Yes
Sweet #3, fruit gum			x	49.7	50.3	Yes
Sweet #4, fruit gum	Raspberry juice		x	50.4	49.6	?
Sweet #5, fruit gum	Raspberry juice		x	51.0	49.0	?
Sweet #6, lollipops			x	50.4	49.6	Yes
Sweet #7, fruit gum			x	50.2	49.8	Yes
Sweet #8, fruit gum			x	50.4	49.6	Yes
Fruit bar #1	Raspberry 1.3%	x		49.2	50.8	No
Yoghurt #1	Raspberry 14%			100	0	Yes
Yoghurt #2	Raspberry 7.5%			100	0	Yes
Yoghurt #3	Raspberry 1.7%	x		49.5	50.5	No
Yoghurt #4	Raspberry 7%	x		100	0	Yes
Yoghurt #5	Raspberry 8%	x		100	0	Yes
Yoghurt #6	Raspberry 6%			100	0	yes

### SPME fibers

SPME fibers were purchased from Supelco, (Supelco, Bellefonte, Pennsylvania, USA), all coated on a fused silica fiber and with a length of 1 cm; divinylbenzene (DVB)/polydimethylsiloxane (PDMS) 65 *μ*m coating, carboxen (CAR)/(PDMS) 75 *μ*m coating, and DVB/CAR/PDMS 50 *μ*m DVB and 30 *μ*m CAR on PDMS coating.

### SPME‐chiral‐GC‐MS method

The SPME fibers were used for extracting aroma compounds from raspberries and subsequent analysis, using automated headspace solid phase micro extraction (HS‐SPME), using a GC‐Combi PAL (CTC Analytical, Zwingen, Switzerland) on an Agilent 6890 gas chromatograph (GC) (Agilent Technologies, Inc., Wilmington, Germany) equipped with an Agilent 5979 Mass Selective Detector.

Samples of fresh raspberries were macerated with a fork prior to transfer to the sample vials. No sample preparation, except homogenization was performed on jam, soda, yoghurt, and dried raspberry samples. Sweets and the fruit bar were cut into 5 mm pieces prior to transfer to the sample vials. Approximately, 4 g of all samples was placed in a 10 mL headspace vial. The vial was heated to 60°C before the SPME fiber was exposed to the headspace. Extraction was carried out at 60°C for 30 min using agitation. Optimized extraction conditions were adapted from Cagliero et al. (2012). Hereafter, the analytes were thermally desorbed into the GC inlet at 230°C for 5 min in splitless mode. The GC was used with the following temperature program: 50°C for 5 min and then raised at 30°C/min to 100°C and raised again at 2°C/min to 145°C and finally 30°C/min to 200°C for 4 min (GC runtime: 35 min). For the method optimizing part, the following temperature program was used: 50°C for 7 min and then raised at 1.5°C/min to 180°C and raised again at 6°C/min to a final temperature of 200°C/min for 5 min (GC runtime: 102 min). The column was a *β*‐DEX 225 (Supelco, USA) consisting of nonbonded 25% 2,3‐di‐O‐acetyl‐6‐O‐TBDMS‐*β*‐cyclodextrin in a SPB‐20 phase 0.25 mm × 30 m, 0.25 *μ*m film thickness. Carrier gas was helium at 0.8 mL/min, 33 cm/sec. The transfer line temperature was 250°C, MS source 230°C, MS Quad 150°C. The mass spectrometer was used in Electron Ionization (EI) mode, using Scan mode (*m/z* 40–400) for the method optimizing part using Nist library for identification of compounds. The mass spectrometer was operated in Single Ion Monitoring (SIM) for food samples, using *m/z* 121 as quantifier ion and 192, 136, and 93 as qualifiers to ensure the identity and purity of the peaks. Quality ratio control of the qualifiers were ±20% and quality control of the retention time was ±0.2 min. Samples were analyzed as single determinations, which were deemed sufficient because percentages of R‐*α*‐ionone close to 100 in the samples would indicate natural flavor whereas values close to 50 would indicate a synthetic flavor.

## Results and Discussion

**Figure 1:**
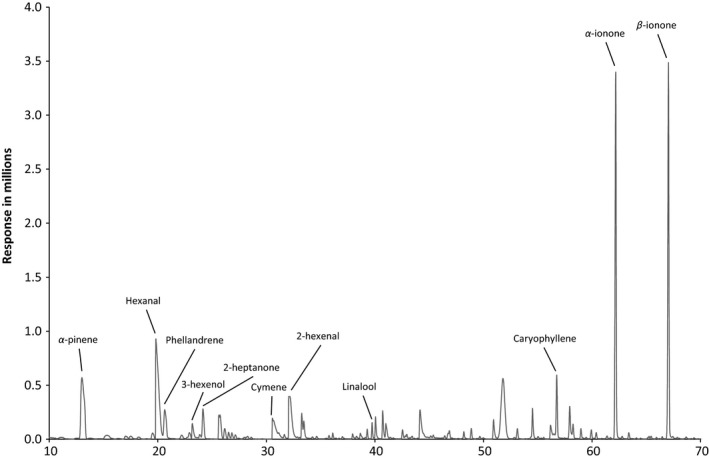
Full scan head space SPME GC‐MS chromatogram of macerated raspberries, GC runtime 102 min.

Before analyzing the samples, the analytical method was optimized in order to obtain a fast and reliable method for the documentation of the authenticity of raspberry flavor in food products. The optimization was performed using macerated raspberries as samples and included SPME fiber‐coating selection and the influence of addition of sodium chloride to the samples. A full scan SPME GC‐MS chromatogram of macerated raspberries, using a DVB/CAR/PDMS fiber is shown in Figure 1, where it appears that *α*‐ionone and *β*‐ionone are the highest peaks.

**Figure 2:**
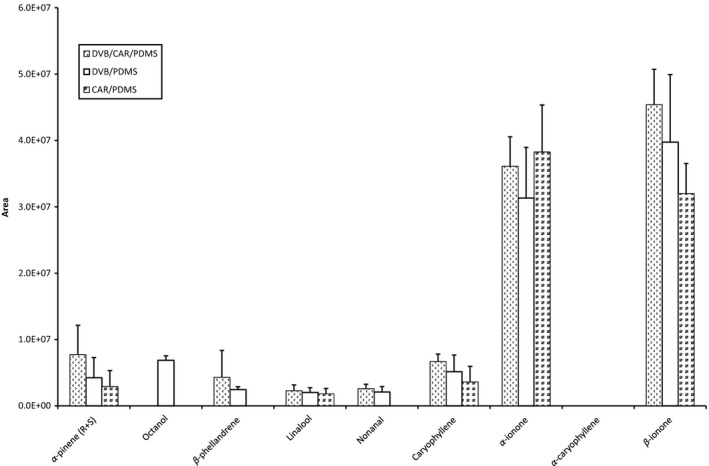
Peak areas for eight analytes extracted from raspberries with DVB/CAR/PDMS, DVB/PDMS, and CAR/PDMS SPME fibers.

To study the influence of fiber coating on the SPME the following fiber types were selected, all coated on a fused silica fiber and with a length of 1 cm; divinylbenzene (DVB)/polydimethylsiloxane (PDMS) 65 *μ*m coating, carboxen (CAR)/(PDMS) 75 *μ*m coating and DVB/CAR/PDMS with 50 *μ*m DVB coating/30 *μ*m CAR on PDMS coating. Macerated raspberries were used as a basis model for the experiments. For this method optimizing study, only the ability to extract analytes from raspberries was investigated and the levels were therefore not quantified but based on comparison of peak areas and presented in Figure 2.

The three SPME fibers showed approximately similar abilities to extract the seven major chemical constituents from raspberry headspace. With the exception that octanol was not extracted by DVB/CAR/PDMS and CAR/PDMS. In addition *β*‐phellandrene and nonanal were not extracted by CAR/PDMS. The DVB/CAR/PDMS fiber showed slightly better extraction efficiency for ionone and was therefore selected for the further studies.

### Influence of addition of sodium chloride to samples

**Figure 3:**
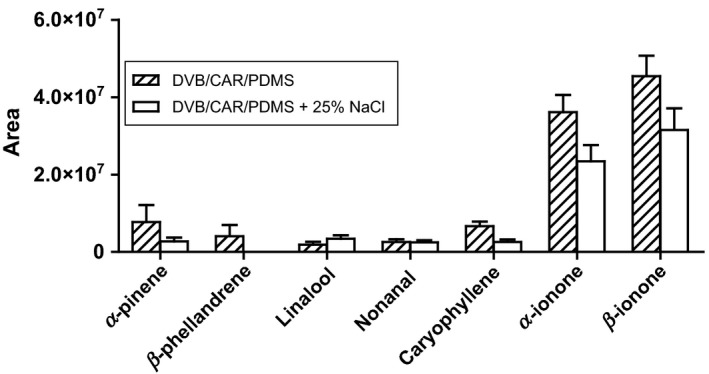
Peak area for seven analytes extracted from raspberries with DVB/CAR/PDMS SPME fiber with and without addition of NaCl.

The effect of addition of sodium chloride to the matrix was investigated, as increased ionic strength usually improves the extraction efficiency of hydrophilic compounds (Kudlejova et al. 2012). Sodium chloride was added to the sample matrices to a concentration of 25 w/w % and analytes extracted with a SPME fiber coated with DVB/CAR/PDMS and presented in Figure 3.

From Figure 3 it is clear that addition of sodium chloride does not have a positive effect upon extraction of the majority of the analytes from raspberries. On the contrary it looks like the salt has a negative effect on the extraction especially for *α*‐pinene, caryophyllene, *α*‐ and *β*‐ionone. For the other compounds analyzed the effect was less pronounced. Only the extraction of linalool was a little higher when sodium chloride was added. Pawliszyn 2012 reported that the positive effect of salt increases with increased polarity of the analytes, which is in agreement with the results found in this study (Pawliszyn 2012).

The result for *β*‐ionone is in agreement with the results of Yang and Peppard, who found that the extraction of *β*‐ionone decreases with higher sodium chloride concentrations when extracted with a SPME fiber, coated with 100 *μ*m PDMS (Yang and Peppard 1994).

### Content of R‐(E)‐*α*‐ionone and S‐(E)‐*α*‐ionone in foods, beverages and sweets from the Danish market

**Figure 4:**
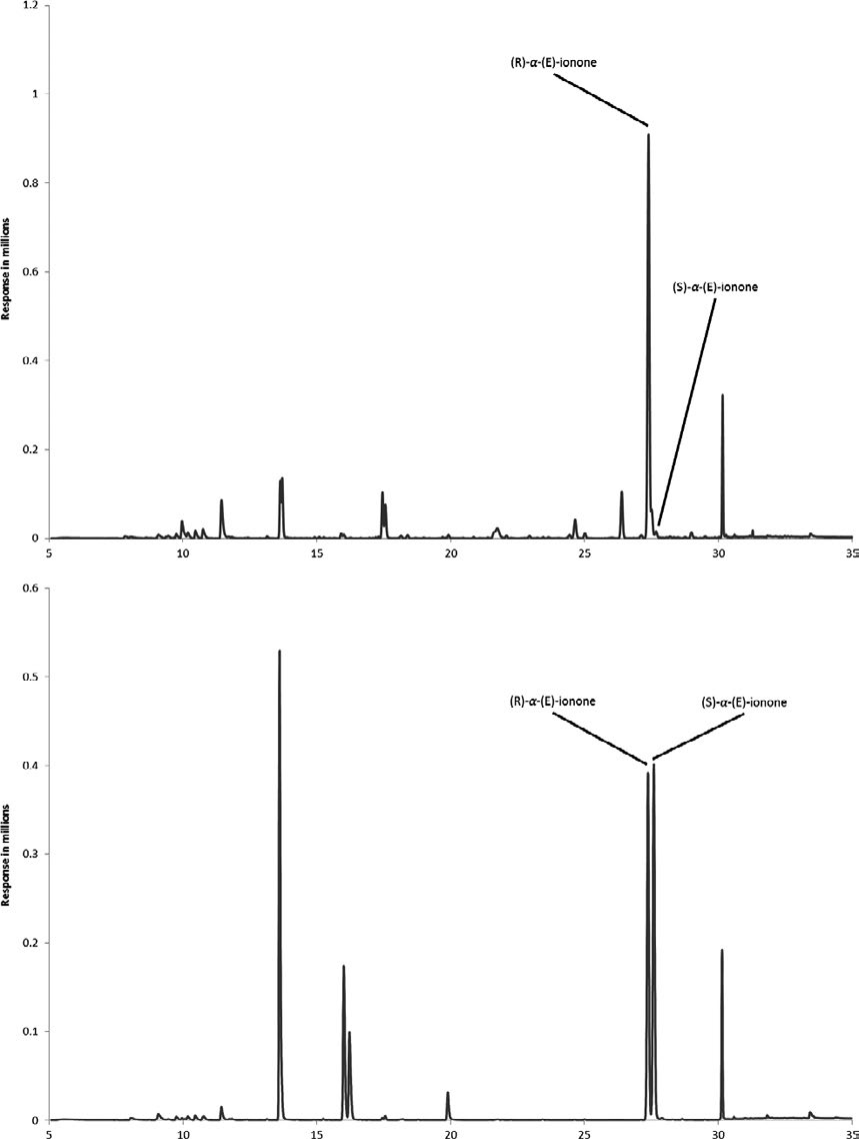
Chromatograms of raspberry jam containing primarily R‐(E)‐*α*‐ionone (top) and of yoghurt containing synthetic raspberry aroma with both R‐(E)‐*α*‐ionone and S‐(E)‐*α*‐ionone (bottom).

For authenticity investigation of raspberry flavored foods, using head space SPME chiral GC‐MS the most important compound is *α*‐ionone. From the chromatogram in Figure 4, it can be seen that (S)‐*α*‐ionone is barely detectable in macerated raspberry (top) compared to the peak of (R)‐*α*‐ionone. This is in accordance with previously published results on the analyses of enantiomer ratios of *α*‐ionone in raspberries showing that (R)‐*α*‐ionone constitutes more than 97% (Sewing et al., 2005; Ravid et al. 2010). On the contrary, synthetic raspberry aroma (Fig. 4 bottom) shows the presence of both enantiomers in almost equal amounts. Synthetic *α*‐ionone can be added to natural raspberry flavor, that is, in order to fortify the flavor and/or reduce price. In that case, the enantiomer ratio will not be 50:50, but reflect the percentage of synthetic aroma added to the natural raspberry aroma and it would still be possible to detect (S)‐*α*‐ionone indicating a not purely natural flavor.

Twenty seven samples of food beverages and sweets from the Danish retail market were analyzed for enantiomer ratio of (E)‐*α*‐ionone using the described head space solid phase microextraction method, including chiral‐gas chromatography combined with mass spectrometric detection, using the SIM mode. Table 1 present the results calculated as the percentage of R‐(E)‐*α*‐ionone and S‐(E)‐*α*‐ionone in all samples. Six samples of raspberry jam declared to contain between 35 and 50% fruit, all had a huge preponderance of the (R) enantiomer of *α*‐ionone with the (S) enantiomer being barely detectable. These jams were all declared not to have any added aroma which the analyses confirmed.

Analyses of two out of five sodas declared to contain natural aroma only, showed that these contained only (R)‐*α*‐ionone which is in accordance with the declared content. The three sodas declared to contain aroma showed an enantiomer ratio of 50%, which is, also, in accordance with the declaration and confirmed that synthetic *α*‐ionone had been added to the products.

The dried raspberries only contained the (R) enantiomer of *α*‐ionone, so no synthetic *α*‐ionone was added to this product. Six of the sweets samples had a 50:50 enantiomer ratio which is in accordance with the declared use of aroma in these samples. Two of the sweets were declared to contain raspberry juice concentrate, 5% as well as aroma. However, the content of ionone from this source must be very low as addition of raspberry juice containing exclusively the (R) enantiomer would have been expected to change the enantiomer ratio from the observed 50:50%. The fruit bar was declared to contain both 1.3% raspberry and natural aroma, however, the measured enantiomer ratio of 50:50% suggest that synthetic aroma was added to this product and accordingly the declaration is not compliant with legislation.

Finally, six yoghurt samples, declared to contain between 1.3% and 14% raspberries, were analyzed. Three of the samples were declared, in addition to raspberries, also to contain the natural aroma. For five of the raspberry yoghurt samples only the (R) enantiomer of *α*‐ionone were found in the products indicating natural raspberries were used in the product. However, for yoghurt #3 declared to contain 1.7% raspberry and natural aroma, a 50:50 enantiomer ratio for R‐*α*‐ionone and S‐*α*‐ionone was observed indicating that synthetic *α*‐ionone was added as aroma to this product contrary to the declared use of natural aroma.

## Conclusions

A fast and simple headspace SPME‐chiral‐GC‐MS method for analyses of authenticity of l raspberry flavor in foods has been developed and used to analyze samples of jams, sodas, sweets, fruits bars, dried raspberries and yoghurts. Raspberry jam, dried raspberries, and sodas declared to contain natural aroma all contained almost only R‐*α*‐ionone supporting the content of only naturally raspberry or naturally raspberry aroma used in the products. All sweets tested had an almost equal distribution between R‐*α*‐ionone and S‐*α*‐ionone indication that synthetic aroma was added to these products in agreement with the information from the declaration. For two out of the 27 products tested, the fruit bar and one of the raspberry yoghurts, both R‐*α*‐ionone and S‐*α*‐ionone was detected at an enantiomer ratio of 50% indicating the use of synthetic aroma in the products, which is in disagreement with the declared information on the products.

## Conflict of Interest

None declared.
